# Leukemoid Reaction in a Severe COVID-19 Patient

**DOI:** 10.7759/cureus.17865

**Published:** 2021-09-10

**Authors:** Diogo R Sene, Diego M Watashi

**Affiliations:** 1 Intensive Care Unit, Hospital Dr. Arnaldo Pezzuti, Mogi das Cruzes, BRA; 2 Critical Care Medicine, Universidade de Mogi das Cruzes, Mogi das Cruzes, BRA

**Keywords:** covid-19, leukocytosis, case report, leukemoid reaction, sars-cov-2

## Abstract

Leukemoid reaction is defined by a leukocyte level above 50 x 10^3^/µL with a predominance of mature neutrophils and the presence of immature granulocytic forms in the peripheral blood (left shift). We report a case of a 36-year-old woman with severe coronavirus disease 2019 (COVID-19) infection admitted to the ICU with a leukocytosis of 70.9 x 10^3^/µL white blood cells (WBC) throughout her hospitalization. A left shift with bandemia along with toxic granulations was also noticed and further investigation excluded more commonly known causes. A presumptive diagnosis of leukemoid reaction was made secondary to COVID-19 infection; however, it could not be confirmed since workup for lymphoproliferative disorders could not be performed as the patient passed away. The leukemoid reaction could be associated with severe COVID-19 infection; however, more data are needed to evaluate this association.

## Introduction

Leukemoid reaction (LR) is defined by a leukocyte count above 50 x 10^3^/µL with a predominance of mature neutrophils and the presence of immature granulocytic forms in the peripheral blood (left shift). It is caused by inflammatory reactions or organic stress with different etiologies: infections, drugs, intoxications, hemorrhage, and malignancy [1].

Since the first coronavirus disease 2019 (COVID-19) reports, the hematologic manifestations were described in most cases as normal or decreased leukocyte count and lymphopenia, and less commonly, leukocytosis. This case report describes a rare presentation of leukemoid reaction in a COVID-19 patient [2].

## Case presentation

A 36-year-old woman presented to the emergency department reporting worsening shortness of breath associated with malaise and headaches. Four days prior, the patient tested positive for COVID-19 infection at a primary care facility and was prescribed azithromycin and prednisone for five days. On examination at the emergency department, she was in respiratory distress. Her vital signs were: temperature 37.9°C, oxygen saturation 85% on room air, heart rate 112/minute, and respiratory rate 31/minute. There were no other positive findings on her physical examination. Her past medical history included obesity (BMI of 35.7 kg/m^2^), diabetes, and hypertension for which she was non-compliant with the treatment.

Initial blood workup showed elevated inflammatory markers (C-reactive protein: 55.9 mg/dl, erythrocyte sedimentation rate [ESR]: >100 mm/hr, ferritin: 3014 ng/mL, and elevated D-Dimer: 1.94 mg fibrinogen equivalent units [FEU]/L). Respiratory alkalosis was observed on arterial blood gas analysis and bilateral ground-glass infiltrates were noted on chest CT.

Despite being on oxygen support by face mask at 15L/min, her symptoms rapidly worsened requiring intubation and mechanical ventilation. She was transferred to the medical intensive care unit (ICU) for proper care. During her stay in the ICU, the patient was started on dexamethasone 6 mg per day for 10 days and prophylactic enoxaparin and was ventilated in accordance with the ARDSnet (Acute Respiratory Distress Syndrome Network) guide [3].

On the following days in the ICU, the patient’s condition deteriorated showing prolonged capillary refill time and low blood pressure. Fluid resuscitation and further norepinephrine were initiated as well as empiric antibiotic therapy with meropenem was started. Routine infection workup including blood, respiratory, and urine cultures was requested. 

Laboratory findings showed marked leukocytosis with left shift (white blood cells [WBC] 27.8 x 10^3^/µL with absolute neutrophil count [ANC] 20.2 x 10^3^/µL), and normocytic anemia (hemoglobin of 9.4 gm/dL, mean corpuscular volume [MCV] 82.9, mean corpuscular hemoglobin [MCH] 27.3) and lactate of 3 mmol/L were observed.

**Figure 1:**
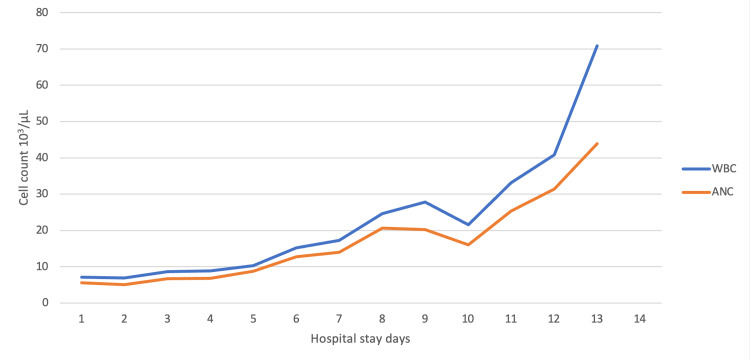
White blood cells and absolute neutrophil values during the hospital stay. WBC, white blood cells; ANC, absolute neutrophil count.

Subsequent laboratory investigations showed persistent leukocytosis with left shift (WBC 70.9 x 10^3^/µL and ANC 44.01 x 10^3^/µL), metamyelocytes (9%), myelocytes (6%), mild lymphocytosis (5.67 x 10^3^/µL), monocytosis (2.13 x 10^3^/µL), and toxic granulations (Figure 1). Blood, tracheal aspirate, central venous catheter tip, and urine culture results were negative and the antibiotic therapy was stopped. A thorough physical exam showed no positive findings. Procalcitotin was lower than 0.1 ng/mL, leukocytic alkaline phosphatase score was 170, uric acid level was 7.1 mg/dL, vitamin B12 level was 183 pg/mL, and folic acid level was 5.4 ng/L. Moreover, there were no positive chest X-ray findings. Further workup for the lymphoproliferative disorder was not possible as the patient passed away in the hospital on day 14.

## Discussion

LR is caused by an inflammatory stimulus that originated from outside of the bone marrow. The most common causes are infections like *Clostridium difficile*, disseminated tuberculosis, and shigellosis. Other associated conditions are exposure to drugs (corticosteroids, minocycline, and recombinant hematopoietic growth factors), ethylene glycol intoxication, malignancy (carcinoma, melanoma, Hodgkin’s lymphoma, and sarcoma), and hemorrhage [1,4].

LR share close features with leukemia, requiring the exclusion of chronic myelogenous leukemia and chronic neutrophilic leukemia in all cases. Many tests may distinguish those etiologies, including white blood cells count with differential count, peripheral blood smear, leukocyte alkaline phosphatase (LAP) score, serum vitamin B12, bone marrow aspiration, cytogenetic studies, immunophenotyping of peripheral blood and bone marrow, serum levels of hematopoietic growth factors, and clonality studies of blood neutrophils. The diagnosis of LR is made by the presence of mature neutrophilia in the peripheral blood smear with a left shift without known underlying conditions and excluding leukemia [4,5].

The presence of an LR should first raise concern for infection, especially in patients exposed to high infection risk procedures or housing, as an ICU. Therefore, a thorough investigation must be conducted to exclude this diagnosis.

Corticosteroids are a well-known cause of increasing WBC counts [6,7]. Increases in WBC counts of an average of 4 x 10^3^/µL have been reported; however, it may vary, mostly related to the dose of glucocorticoid [8]. Moreover, glucocorticoid-induced leukocytosis is generally not associated with worsening in the condition that is being appropriately treated and the left shift is not as pronounced as it is in infection [9].

The treatment of LR should be guided by the underlying etiology, and a normalization of the leukocyte count is expected after the etiology resolution [5].

While a rare finding, few case reports show the presence of LR in patients with severe COVID-19. Similar case reports of LR with COVID-19 are presented in Table 1.

**Table 1:** Similar case reports of COVID-19 associated with leukemoid reactions. WBC, white blood cell count; PBS, peripheral blood smear; LAP, leukocytic alkaline phosphatase; FISH, fluorescence in situ hybridization; JAK2, Janus kinase 2; CALR, calregulin gene; MPL, thrombopoietin receptor gene.

	Gender and age of the patient	Highest WBC count and complete blood count	Lab tests	Outcome
Our case	Female, 36 years	WBC count 70.9 x 10^3^/µL. Normocytic anemia and normal platelets.	PBS: neutrophilic leukocytosis with left shift, metamyelocytes, myelocytes, mild lymphocytosis, monocytosis, and toxic granulations. LAP 170.	Passed away on day 14 of hospitalization.
Tabassum et al. [5]	Male, 76 years	WBC count 96.6 x 10^3^/µL. Normal hemoglobin and platelets.	PBS: neutrophilic leukocytosis with left shift and presence of myelocytes and metamyelocytes. JAK2 negative. LAP 380.	Passed away on day five of hospitalization.
Tarekegn et al. [1]	Female, 64 years	WBC count 95.9 x 10^3^/µL. Normocytic anemia and thrombocytosis.	PBS: neutrophilia and left shift with many bands with toxic granulations, metamyelocytes, and myelocytes. Cytometry negative for the lymphoproliferative disorder. FISH negative for BCR/ABL, JAK2, CALR, and MPL mutations.	Passed away on day 13 of hospitalization.

All reports describe a poor outcome, with the patients passing at different moments of the disease. LR has been reported to be a high mortality indicator in infectious etiologies [10]. Recent literature suggests a correlation between neutrophilia and disease severity in COVID-19 [11]. But the lack of data cannot confirm an association between LR in COVID-19 and disease severity or mortality. Workup for lymphoproliferative disorders was not performed due to the unfortunate death of this patient, creating uncertainty in the diagnosis of LR secondary to COVID-19.

## Conclusions

The diagnosis of LR in a COVID-19 patient should be considered facing extremely high WBC levels. Other severe conditions should be excluded as an etiology of the LR. More data are needed to study any prognostic value of LR in COVID-19.

## References

[1] Tarekegn K, Colon Ramos A, Sequeira Gross HG, Yu M, Fulger I (2021). Leukemoid reaction in a patient with severe COVID-19 infection. Cureus.

[2] Zhu J, Ji P, Pang J (2020). Clinical characteristics of 3062 COVID-19 patients: a meta-analysis. J Med Virol.

[3] (2021). NIH NHLBI ARDS clinical network mechanical ventilation protocol summary. http://www.ardsnet.org/files/ventilator_protocol_2008-07.pdf.

[4] Sakka V, Tsiodras S, Giamarellos-Bourboulis EJ, Giamarellou H (2006). An update on the etiology and diagnostic evaluation of a leukemoid reaction. Eur J Intern Med.

[5] Tabassum SA, Bibi T, Tariq F (2020). Unusal leukemoid reaction in a COVID-19 patient: a case report. Biol Clin Sci Res J.

[6] Abramson N, Melton B (2000). Leukocytosis: basic of clinical assessment. Am Fam Physician.

[7] Nakagawa M, Terashima T, D'yachkova Y, Bondy GP, Hogg JC, van Eeden SF (1998). Glucocorticoid-induced granulocytosis: contribution of marrow release and demargination of intravascular granulocytes. Circulation.

[8] Dale DC, Fauci AS, Guerry D IV, Wolff SM (1975). Comparison of agents producing a neutrophilic leukocytosis in man. Hydrocortisone, prednisone, endotoxin, and etiocholanolone. J Clin Invest.

[9] Shoenfeld Y, Gurewich Y, Gallant LA, Pinkhas J (1981). Prednisone-induced leukocytosis. Influenced of dosage, method and duration of administration on the degree of leukocytosis. Am J Med.

[10] Portich JP, Faulhaber GA (2020). Leukemoid reaction: a 21st-century cohort study. Int J Lab Hematol.

[11] Huang G, Kovalic AJ, Graber CJ (2020). Prognostic value of leukocytosis and lymphopenia for coronavirus disease severity. Emerg Infect Dis.

